# Placement Of A Coronary Sinus Pacing Lead From A Sub-occluded Left Subclavian Vein Using A Collateral Vein To The Right Subclavian Vein

**Published:** 2011-11-15

**Authors:** Marco Brieda, Luca De Mattia, Ermanno Dametto, Federica Del Bianco, Gianluigi Nicolosi

**Affiliations:** Department of Cardiology ARC, Azienda Ospedali Riuniti del Pordenonese, Pordenone, Italy

**Keywords:** Resynchronization therapy, Vein occlusion, Collateral circulation, Pacemaker upgrading

## Abstract

Upgrading of a pacing system in the presence of a subclavian occlusion is technically challenging. We describe the case of a patient who underwent a successful upgrading procedure of an implantable cardioverter-defibrillator (ICD) to a biventricular defibrillator (ICD-CRT) in the presence of a suboccluded left subclavian vein, using a collateral vein that drained into the contralateral subclavian vein.

## Introduction

Patients with an existing implantable cardioverter-defibrillator (ICD) may benefit from an upgrade to a biventricular cardioverter-defibrillator (ICD-CRT). Upgrading to ICD-CRT in the presence of a subtotal subclavian occlusion is technically challenging. Nevertheless, every effort has to be made to complete the procedure on the same side of the first implant, in order to preserve the contralateral venous system.

## Case report

**Figure 1:**
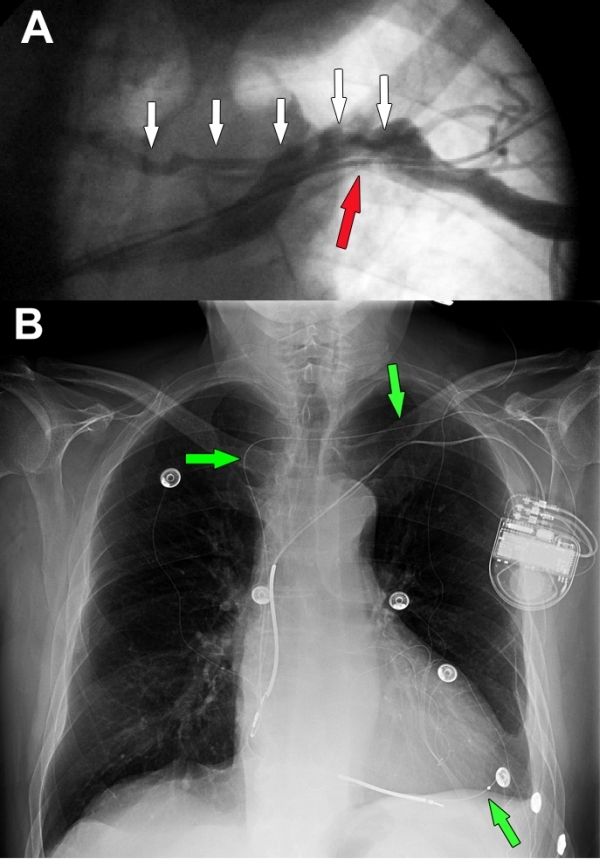
A. Venogram, performed with contrast injection in the left basilic vein, demonstrating a suboccluded left subclavian vein (red arrow) and the presence of a collateral vein (white arrows) draining into the right subclavian vein. B. Chest radiograph (anteroposterior view) obtained the day after the procedure. The coronary sinus pacing lead (green arrows) passes from the left subclavian vein to its final position via a collateral vein and the right subclavian vein. The CS lead is placed in a posterior location.

A 78-year old man with a history of dilated cardiomyopathy, left ventricular ejection fraction 30% and New York Heart Association class III heart failure refractory to medical therapy, was referred for upgrading of a dual-chamber ICD to ICD-CRT (both leads were previously inserted from the left subclavian vein). The patient was pacemaker dependent. A pre-implant venography showed a suboccluded left subclavian vein (Figure 1A, red arrow) and a collateral vein which ran over the subclavian vein (Figure 1A, white arrows) and drained into the contralateral subclavian vein. Following vascular access, multiple attempts to cross the obstruction with a guidewire were unsuccessful. A 0.035-in J-tip hydrophilic guidewire was then successfully advanced from the collateral vein to the right subclavian vein, the superior vena cava and then the right atrium. A delivering system composed of a 7 French, 50-cm long catheter with hydrophilic coating (Medtronic, St Paul MN, product number 6250-MB2X) and a 5.5 French vein selector inner catheter was advanced over the guidewire into the coronary sinus (CS) ostium and then guided into a posterior branch of the CS. Finally, a 4 French pacing lead (Medtronic, St Paul, MN, product number 4196) was successfully deployed into the CS branch with optimal pacing and sensing thresholds and no diaphragmatic stimulation at high outputs. With biventricular pacing the QRS duration shortened from 140 to 105 msec. The day after a chest X-ray confirmed the correct position of the CS lead (Figure 1B, green arrows) and the patient was discharged.

## Discussion

Obstruction of the access vein occurs frequently in patients with implanted pacing systems. Previous reports in pacemaker patients found an incidence of asymptomatic high grade stenosis-occlusion ranging from 20% up to 30%, whereas symptomatic cases occurred less frequently (1-3%) [1]. Other reports examining ICD patients showed similar rates (13-25%) of asymptomatic vein subocclusion-occlusion. [1,2] 

Risk factors for upper vein thrombosis in pacemaker patients are not clearly defined: nonetheless the presence of multiple leads, absence of anticoagulation therapy, personal history of venous thrombosis, use of hormone therapy, low left ventricular ejection fraction and previous transvenous temporary pacing leads seem to play a role, whereas age, sex, body size, site of access, lead polarity, insulation and calibre and time from implant do not appear to influence the incidence of vein  thrombosis. [3] Specific risk factors for development of vein thrombosis in ICD patients are a history of pacemaker implantation prior to the ICD system and the presence of dual shocking coil leads. [3]

Upgrading of the pacing system in the presence of a subtotal subclavian occlusion is technically challenging. Other authors reported the insertion of a defibrillator lead through a collateral vein in the presence of an occluded subclavian vein. [4,5] To the best of our knowledge this is the first report of a CS lead implant using a collateral vein. In this patient a pacing lead and an introducer with the smallest diameter available were used to avoid damaging the collateral vein, along with extreme caution and smoothness in pushing and torquing the delivering system to reach the CS ostium. Furthermore, the introducer's hydrophilic coating made easier for the delivering system to slide through the collateral vein.

Other authors previously described alternative techniques to implant CS pacing leads in the presence of a subclavian vein obstruction. Pires and colleagues reported of CS lead placement via the internal jugular vein [6]. The CS pacing lead however had to be tunnelled over the clavicle to the ICD pocket, thus exposing the patient to the potential risk of lead dislodgement or fracture due to the clavicular movement. Upgrading to biventricular pacing using the supraclavicular puncture of the subclavian [7] or innominate [8] vein in patients with a pre-existing ICD were also described. As with the supraclavicular approach, tunnelling of the lead over the clavicle was needed. Possible related complications include pneumothorax and puncture of the brachiocephalic trunk or the ascending aorta. Vein recanalization by venoplasty [9] (if guide wire access is achievable beyond the occlusion) or lead extraction [10] carries up to a 1.6-2% risk of major complications. A more medial approach for subclavian vein puncture is another feasible option, but could expose the patient to a higher risk of developing pneumothorax or lead fracture.

## Conclusions

In case of a subclavian obstruction the placement of a CS pacing lead via a collateral vein (where present and adequate in size) might be considered as a feasible option before attempting alternative and perhaps riskier approaches.
